# Assessing risk factors for tuberculosis treatment failure among pulmonary tuberculosis patients in four health regions of Burkina Faso, 2009

**DOI:** 10.11604/pamj.supp.2018.30.1.15792

**Published:** 2018-05-15

**Authors:** Bernard Sawadogo, Mamadou Sawadogo

**Affiliations:** 1 West Africa Field Epidemiology and Laboratory Training Program, Burkina Faso; 2 University of Ouagadougou-I, Burkina Faso

**Keywords:** Risk factor assessment, tuberculosis, treatment failure, pulmonary tuberculosis, Burkina Faso

## Abstract

Conducting research studies is one of the competencies that epidemiology students should have after two years training as a crucial component to identifying and resolving public health problems. This case study is developed from a research study on assessing risks factors on tuberculosis treatment failure in Burkina Faso in 2009 to help students to cover these competences. This case study is ideal for reinforcing principles of conducting case-control studies for risk-factor assessment. The case study will help epidemiology learners outline a study proposal, reinforce knowledge about research design, data collection and data analysis issues, recognize the importance of ethics in research, and interpret results and use them to make conclusions and recommendations.

## How to use this Study

**General instructions:** ideally 1 to 2 instructors facilitate the case study for 15 to 20 trainees in a classroom. The instructor should direct participants to read the paragraphs out loud, going around the room to give each participant a chance to read. When the participant reads a question, the instructor directs all participants to perform calculations, construct graphs, or engage in discussions. The instructor may direct the class to play different roles or take different sides in answering a question. As a result, participants learn from each other, not just from instructors. Specific instructor’s notes are included with each question in the instructor’s version of this case study.

**Audience:** learners in epidemiology and other public health practitioners who are interested in this topic

**Prerequisites:** before using this case study, participants should have received lectures on study design, data management, and protocol writing.

**Materials needed:** Laptop with Microsoft Word with Excel, Power Point and Epi Info, graph paper, flipchart or white board with markers

**Level of training and associated public health activity:** FETP Resident 2nd years – conducting research study

**Time required:** 2-3 hours

**Language:** English

## Competing interest

The authors declare no competing interest.
